# Effects of Activated Charcoal on Growth, Immunity, Oxidative Stress Markers, and Physiological Responses of Nile Tilapia Exposed to Sub-Lethal Imidacloprid Toxicity

**DOI:** 10.3390/ani11051357

**Published:** 2021-05-11

**Authors:** Samah A. A. Abd El-hameed, Samar S. Negm, Nahla E. M. Ismael, Mohammed A. E. Naiel, Mohamed Mohamed Soliman, Mustafa Shukry, Hany M. R. Abdel-Latif

**Affiliations:** 1 Fish Health and Management Department, Central Laboratory for Aquaculture Research (CLAR), Agriculture Research Center, Abbassa, Abu Hammad, Sharkia 44661, Egypt; samahbasha222@yahoo.com; 2 Fish Biology and Ecology Department, Central Laboratory for Aquaculture Research (CLAR), Agriculture Research Center, Abbassa, Abu Hammad, Sharkia 44661, Egypt; sss.negm1984@gmail.com (S.S.N.); nahla_aquaecology@yahoo.com (N.E.M.I.); 3 Department of Animal Production, Faculty of Agriculture, Zagazig University, Zagazig 44511, Egypt; mohammednaiel.1984@gmail.com; 4 Clinical Laboratory Sciences Department, Turabah University College, Taif University, P.O. Box 11099, Taif 21944, Saudi Arabia; mmsoliman@tu.edu.sa; 5 Department of Physiology, Faculty of Veterinary Medicine, Kafrelsheikh University, Kafrelsheikh 33516, Egypt; mostafa.ataa@vet.kfs.edu.eg; 6 Department of Poultry and Fish Diseases, Faculty of Veterinary Medicine, Alexandria University, Alexandria 22758, Egypt

**Keywords:** *Oreochromis niloticus*, antioxidant, hematology, nitric oxide, lysozyme

## Abstract

**Simple Summary:**

Finding a suitable feed supplement is important for maintaining fish health and sustainability of the aquaculture industry. From these supplements, research studies have shown that activated charcoal (AC) has been extensively used for veterinary and aquaculture objectives as a “Universal Antidote” against several toxicants and aquatic pollutants. Therefore, the mitigating roles of dietary supplementation with different AC levels on physiological responses of Nile tilapia exposed to sub-lethal imidacloprid (IMID) toxicity were evaluated. The findings of this study revealed that dietary supplementation with 14.30 g AC/kg diet positively modulated the toxic impacts of IMID-intoxicated fish.

**Abstract:**

The existing study was designed to assess the influences of dietary activated charcoal (AC) on the growth performance, immune responses, antioxidative status, and its mitigating roles against the physiological responses of Nile tilapia exposed a sub-lethal dose of a neonicotinoid agriculture pesticide, namely, as imidacloprid (IMID). Nile tilapia juveniles were fed on diets supplemented with graded AC levels as 0 (control), 5, 10, 15, and 20 g/kg diet for eight weeks. Growth, hemato-biochemical indices, and antioxidant and immune responses of fish in all groups were evaluated at the end of the feeding experiment. Afterward, fish in all experimental groups were subjected to a sub-lethal dose of IMID (0.0109 μg/L) for two weeks. Then, fish mortalities, stress indicators, and IMID residual levels in liver and flesh were examined. Results of the feeding experiment showed that total feed intake, weight gain, final body weights, and feed efficiency ratio were significantly increased in all AC groups compared with the control group. The survival rate was 100% in all experimental groups. No statistical differences were observed in the hematological picture of all experimental groups except the lymphocyte count, which was significantly increased in all AC groups compared to the control group. Total protein, albumin, globulin, nitric oxide levels, lysozyme, and respiratory burst activities were significantly increased in all AC groups. Serum alanine transaminase, aspartate transaminase, alkaline phosphatase activities, and malondialdehyde (MDA) levels were significantly decreased in all AC groups compared with the AC0 group. After exposure to a sub-lethal dose of IMID, survival rates were significantly elevated, and IMID residual levels in liver and flesh were significantly decreased in all AC groups than in the control group. Moreover, second-order polynomial regression showed that dietary supplementation with 14.30 g AC/kg diet resulted in the lowest blood glucose and serum MDA levels. Conclusively, we suggest dietary supplementation with 14.30 g AC/kg diet to modulate physiological responses of Nile tilapia to sub-lethal IMID toxicity.

## 1. Introduction

Activated charcoal (AC) is an odorless, tasteless, and very fine black powder that acts as an “adsorbent ” for toxicants, gases, poisons, and several impurities [1]. It has been widely applied for medicinal, veterinary, and aquatic medical purposes as a “universal antidotal treatment” for several poisons and environmental toxicants [2]. The mechanisms of actions of AC showed its potential in vitro affinity in the adsorption and elimination of several toxicants such as aflatoxins [3] and pesticide tissue residues [4]. The detoxifying properties of AC have been attributed to its physical and chemical properties, such as its pore size, surface area, and adsorption capability [5]. In terrestrial animals, dietary supplementation with AC has been used to absorb ammonia and nitrogen, improve the functions of the gastrointestinal tract (GIT), and eliminate the impurities and poisonous materials from the GIT [6,7,8].

Studies on Nile tilapia (*Oreochromis niloticus*) showed that optimal dietary levels of AC could improve the health status, fillet composition [2], growth performance, and intestinal histomorphology [9], boost the antioxidant capacity, and reduce the tissue bioaccumulation after environmental heavy metal exposure [10]. Moreover, reports showed that AC could enhance the growth of African catfish (*Clarias gariepinus*) [11], decrease heavy metal bioaccumulation in tissues of big sturgeon (*Huso huso*) [12], and improve the intestinal histomorphology of giant trevally (*Caranx ignobilis*) [13,14].

Other AC forms, for instance, dietary supplementation with bamboo charcoal, considerably enhanced the growth of Tiger puffer fish (*Takifugu rubripes*) [15] and Japanese flounder (*Paralichthys olivaceus*) [16] and reduced the percentage of nitrogen and ammonia in rearing water of Striped catfish (*Pangasianodon hypophthalmus*) [17]. Other studies showed that dietary supplementation with charcoal and wood vinegar mixture improved the body composition analysis of *P. olivaceus* [18]. Moreover, commercial wood charcoal could reduce the environmental load in the rearing water of red tilapia (*Oreochromis* sp.) [19] and enhance the water quality of gilthead seabream (*Sparus aurata*) [20].

Imidacloprid (IMID) (as a neonicotinoid pesticide) has been extensively used for insect and pest control, especially those affecting crops [21]. Although Tišler et al. [22] showed that IMID was steady in the water and did not quickly undergo biodegradation in the ecosystems, the unsafe and unhygienic disposal of IMID will subsequently provoke serious toxic impacts on the exposed organisms [23]. In Nile tilapia, previous studies reported that IMID exposure induced genotoxicity [24], hematological changes [25], histopathological alterations [26,27], oxidative stress, and growth depression [28]. Furthermore, IMID induced neurotoxicity in zebrafish (*Danio rerio*) and rainbow trout (*Oncorhynchus mykiss*) [29,30], oxidative stress injury and genotoxicity in Streaked prochilod (*Prochilodus lineatus*) [31], spinal cord malformations of the Japanese rice fish (*Oryzias latipes*) [32], and genotoxicity and immunotoxicity in Chinese rare minnows (*Gobiocypris rarus*) [33].

Our previously published studies reported the potential efficacy of dietary supplementation with vitamin C, chitosan nanoparticles (ChNPs) [28], and, recently, clinoptilolite and ChNPs in attenuation of sub-acute IMID toxicity in Nile tilapia [34]. The present study aimed to evaluate dietary supplementation effects with AC on growth, hemato-biochemical indices, antioxidant capacity, immunological assays, and modulation of sub-acute IMID toxicity in the exposed Nile tilapia juveniles.

## 2. Materials and Methods

### 2.1. Fish and Rearing Conditions

Two hundred healthy Nile tilapia (*Oreochromis niloticus*) juveniles were procured from the governmental hatchery (Central Laboratory for Aquaculture Research (CLAR), Abbassa, Egypt). Laboratory experiments were achieved at the Fish Wet Laboratory (Department of Fish Biology and Ecology, CLAR, Egypt).

Fish were maintained in the indoor fiberglass tanks for two weeks to be acclimated to the laboratory conditions with 12 Light photoperiod. Each tank was equipped with dechlorinated, fresh tap water supplied with continuous compressed air (through air-stones by using air pumps). Before starting the experiments, fish were fed daily up to apparent satiation on a commercially purchased basal diet (30% crude protein) (Aller-Aqua Co., Egypt). During the acclimation period and thereafter, diets were offered to the fish according to their live weight (3% of their live body weight).

### 2.2. Maintenance of Water Quality Parameters

For maintaining healthy water, one-third of the water (per each aquarium) was siphoned (each two days) to eliminate feces and get rid of the uneaten food particles. The physical and chemical features of the rearing water were biweekly examined throughout the whole experimental period. The mean values of water parameters were maintained as pH (7.50–8.50), un-ionized ammonia (0.03 ± 0.01 mg/L), dissolved oxygen (7.50 ± 0.05 mg/L), nitrite (0.013 ± 0.01 mg/L), and water temperature (27.5 ± 0.5 °C) (these levels were within the suitable ranges necessitated for Nile tilapia).

### 2.3. Experiment I: Feeding Trial

#### 2.3.1. Experimental Diets

The basal commercial diet was obtained to meet the appropriate nutritional requirements for Nile tilapia juveniles. Feed ingredients and chemical composition of the basal diet (%) (on an air-dry basis) (Table 1) were previously published in our study by Abdelghany et al. [35].

Activated charcoal (AC) powder (Sigma-Aldrich, St. Louis, MO, USA) (#161551) (CAS Number 7440–44-0) as decolorizing carbonaceous material with high purity, a molecular weight of 12.01 g mol^−1^, and particle size of –100 (mesh) was used in the current study.

Different graded levels of AC were used and were mixed with the basal diet, whereas five experimental diets were formulated containing 0, 5, 10, 15, and 20 g/kg diet [10]. Diet ingredients were finely ground, and each dose level of AC was then suspended in 100 mL water per kg diet and thoroughly mixed with the other diet ingredients for 40 min using a blender. The mixture was then pelleted using a grinder with a 1-mm diameter paste extruder. All diets were left to dry and then packed into plastic bags and refrigerated at −4 °C until further use.

#### 2.3.2. Experimental Design

Fish (with initial body weight = 33.06 ± 0.74 g) were allocated into five groups known as AC 0 (control), AC 5, AC 10, AC 15, and AC 20. Each group was composed of four replicates (each replicate contained 10 fish per 100-L aquarium) (0.75 × 0.50 × 0.50 m). Fish in each group were fed the corresponding diets for 8 weeks.

#### 2.3.3. Growth Performance

At the end of the feeding experiment (8 weeks), fish were assembled, counted, and group weighed. Fish mortality was documented daily, and dead fish were daily collected. Equations used to evaluate the growth and feed utilization parameters were previously illustrated in Abdel-Latif et al. [36] and Mohammadi et al. [37].

Weight gain (WG) (g) = FBW − IBW(1)

Specific growth rate (SGR, %/day) = 100 [Ln FBW (g) − Ln IBW (g)]/T(2)

where IBW is initial body weight, FBW is final body weight, and T is the rearing period.

Total feed intake (TFI) (g feed/fish) is the summation of the amounts of diets (g) fed to fish in each group throughout the experiment/fish number,

Feed conversion ratio (FCR) = TFI (g)/WG (g)(3)

Feed efficiency ratio (FER) = WG (g)/TFI (g)(4)

Survival rate (SR) (%) = 100 (number of fish at the end of the experiment/number at the start).(5)

#### 2.3.4. Blood Sampling and Serum Separation

By the end of the feeding trial, fish had fasted for 24 h before blood sampling. Eight fish were sampled from each group (*n* = 8) and anesthetized using MS-222 (Argent Chemical Laboratories, Redmond, WA, USA) (200 mg/L). Blood was sampled from the caudal veins and divided into two parts (one part was mixed with anticoagulant into Eppendorf tubes for hematological parameters and the other part was left at room temperature for collection of fish serum). The serum was separated into centrifuge tubes by centrifugation (3000× *g* for 15 min) and stored at −20 °C until being used.

#### 2.3.5. Hematological Indices

Red blood cells’ (RBCs) and white blood cells’ (WBCs) counts were measured by using a hemocytometer [38]. Hemoglobin (Hb) values were measured according to Collier [39]. Hematocrit (HTC) and mean corpuscular hemoglobin concentration (MCHC) values were evaluated as defined by Wintrobe’s method [40]. Mean corpuscular volume (MCV) was assessed by an automated Coulter LH 750 hematology analyzer (Beckman Coulter, Fullerton, CA, USA) [41]. Differential leucocytic counts were calculated according to Klontz’s method [42].

#### 2.3.6. Serum Biochemical Measurements

Serum biochemical indices were measured using colorimetric methods using commercial, fish-specific diagnostic kits (Bio-diagnostic Co. for Modern Laboratory Chemicals, Giza, Egypt). The blood protein profile, including serum total protein (TP) and albumin (ALB) values, were assessed as illustrated by Henry [43] and Wotton and Freeman [44], respectively. Globulin (GLO) values were evaluated from the differences between TP and ALB levels. Serum transaminases such as aspartate aminotransferase (AST) and alanine aminotransferase (ALT) activities in fish sera were assessed according to Reitman and Frankel [45]. Serum alkaline phosphatase (ALP) activity was evaluated by the kinetic assay method [46]. Blood glucose (GLU) concentration was analyzed using specific diagnostic kits (Glu L 1000, PLIVA-Lachema Diagnostika, Brno, Czech Republic) [47].

#### 2.3.7. Antioxidant and Immunological Assays

Nitric oxide (NO) was assessed spectrophotometrically (according to a protocol supported by the manufacturer) by the commercial diagnostic kits (BioChain Institute Inc., Newark, CA, USA). Serum malondialdehyde (MDA) levels (as a marker of lipid peroxidation (LPO)) were calorimetrically assessed by using a commercial diagnostic kit (Lipid peroxide (LPO), OXIS International Inc., Portland, OR, USA) [48]. Respiratory burst activity of the whole blood sample was assessed by nitro blue tetrazolium (NBT) dye [49]. Lysozyme activity (LYZ) was evaluated by using turbidity measurement, described by Siwicki and Studnicka [50] and Ellis [51].

### 2.4. Experiment II: Modulation of Sub-Lethal Imidacloprid Toxicity

#### 2.4.1. Experimental Design

The remaining fish in all experimental groups (32 fish per group) continued feeding on the corresponding diets containing the graded AC levels and then exposed to a sub-lethal dose of imidacloprid (IMID) (Imidacloprid 35% SC) (Tagros Chemicals India Ltd., Chennai, India) for an additional 2 weeks. The selected sub-lethal dose of IMID was one-tenth of the previously calculated 96h LC50 = 0.0109 μg/L. The 96h LC50 of IMID in Nile tilapia was calculated in our study as 0.109 μg/L [28]. To maintain the needed IMID dose, water (in each aquarium) was daily substituted, and the calculated IMID dose was then admixed into a little amount of water before being added to the aquarium water. Dead fish were removed daily and recorded to estimate the mortality rate (MR) (%) and SR (%). Relative percent of survival (RPS) (%) was calculated according to this equation. RPS (%) = 100 × (1 − % of mortality in experimental/% of mortality in control).

#### 2.4.2. Sampling

Serum and tissue (liver and flesh) samples were assembled from eight fish per group (*n* = 8) at the end of the sub-acute toxicity test.

#### 2.4.3. Determination of Serum Stress Biomarkers

Serum MDA levels and blood glucose levels as stress biomarkers were evaluated according to the previously described methods (please see Section 2.3.6 and Section 2.3.7.

#### 2.4.4. Determination of Imidacloprid (IMID) Residues

One gram from the fish tissues (either from liver or flesh from the dorsal muscles) was pooled from each fish (*n* = 12) from each experimental group and then mixed with 5 mL acetonitrile for 4 min.

The tissue homogenate was processed, and IMID residues were determined using high-performance liquid chromatography (HPLC), as in methods previously clarified in Dewangan et al. [52] and Ismael et al. [34].

### 2.5. Statistical Analytics

One-way ANOVA was used to assess the effect of AC after 8 weeks of the feeding experiment. Second-order polynomial regression analysis was done to estimate the optimum dietary AC level for the lowest blood glucose and MDA levels of fish in all experimental groups and exposed to sub-lethal IMID dose for 2 weeks. Differences between experimental groups were clarified using Duncan’s multiple range test as a post hoc test, and *p* < 0.05 was determined as statistically significant. The analyzed data are represented as the mean ± standard error (S.E.). Data analyses were performed using SPSS program version 22 (SPSS, v 22.0; SPSS Inc., Chicago, IL, USA) and GraphPad Prism Software 5.0 (GraphPad Software, Inc., San Diego, CA, USA).

## 3. Results

### 3.1. Results of Experiment I

#### 3.1.1. Growth Performance

Table 2 shows the growth indices and survival rate (SR) (%) of Nile tilapia fed diets supplemented with different AC levels for 8 weeks. There were no significant differences in the initial body weight (IBW) of fish in all groups (*p* > 0.05). TFI was significantly increased (*p* < 0.05) in all AC groups compared to the control group. FBW values were significantly increased in AC5, AC10, and AC15 over AC 0 values. Moreover, WG values were statistically the highest in the AC5 group among all groups. FER values were statistically the highest values in the AC10 group among all groups. FCR was significantly decreased (*p* < 0.05) in all AC groups compared to the control group. SR (%) was 100% in all experimental groups with no recorded mortalities throughout the whole experimental period. This finding suggests that dietary AC supplementation does not induce toxic effects on the treated fish.

#### 3.1.2. Hemato-Biochemical Measurements

There were no significant differences (*p* > 0.05; Table 3) in the hematological picture of fish in all experimental groups, except the lymphocyte counts were significantly increased in all AC-supplemented groups compared with AC 0 group. Moreover, there was a significant increase (*p* < 0.05) in TP, ALB, and GLO values (Table 4) and a significant decrease (*p* < 0.05) in ALP, AST, and ALT activities in all AC groups (Table 4).

#### 3.1.3. Antioxidation and Immune Responses

**Figure 1:**
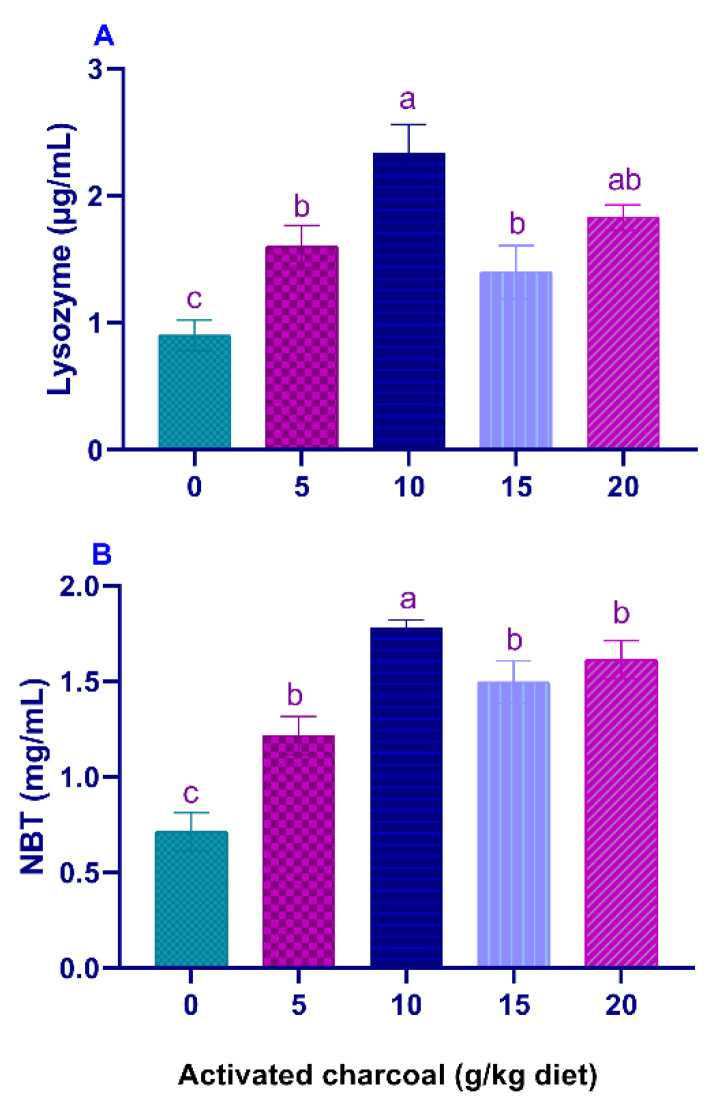
Serum lysozyme (LYZ) activity (**A**) and Nitro blue tetrazolium (NBT) levels (**B**) of Nile tilapia fed diets supplemented with graded, activated charcoal (AC) levels for 8 weeks. (a, b, c) indicate significant differences between groups.

**Figure 2:**
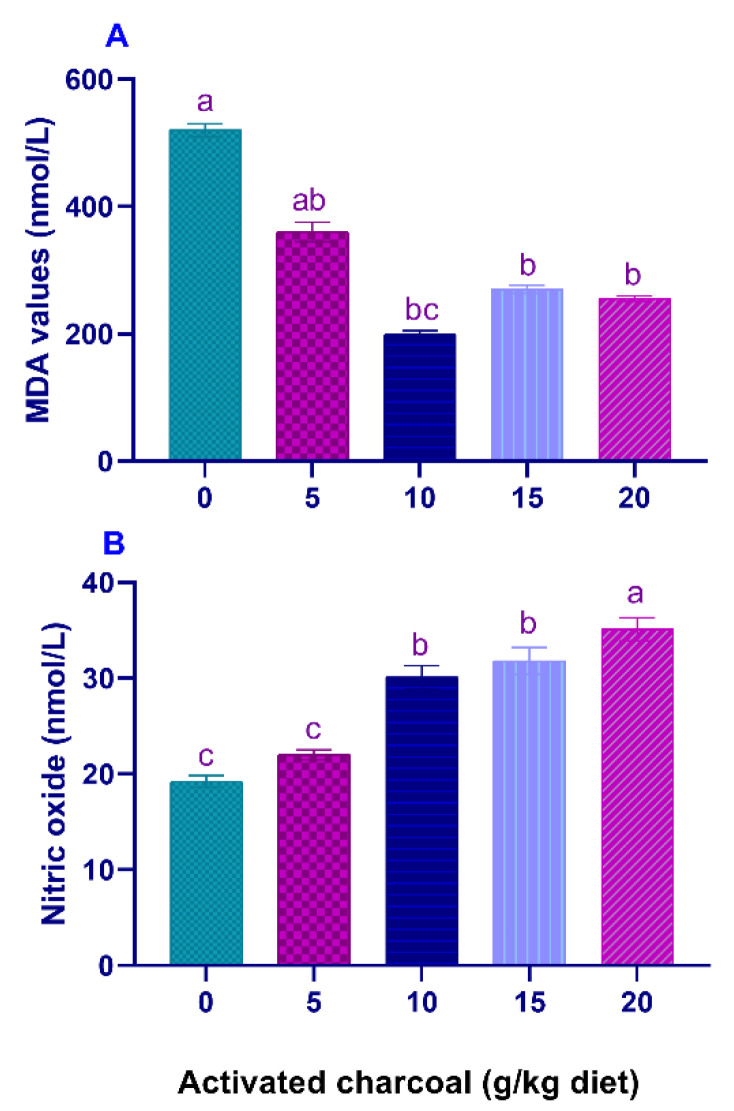
Serum malondialdehyde (MDA) (**A**) and nitric oxide (NO) levels (**B**) of Nile tilapia fed diets supplemented with graded, activated charcoal (AC) levels for 8 weeks. (a, b, c) indicate significant differences between groups.

Serum LYZ activities (Figure 1A) and NBT values (Figure 1B) were significantly elevated (*p* < 0.05) in all AC groups compared to the control group. On the other hand, serum MDA levels (Figure 2A) were significantly lowered (*p* < 0.05) in all AC groups compared to the control group. However, NO levels (Figure 2B) were statistically increased in AC groups compared to the control.

### 3.2. Results of Experiment II: Responses to Sub-Lethal Imidacloprid (IMID) Toxicity

**Figure 3:**
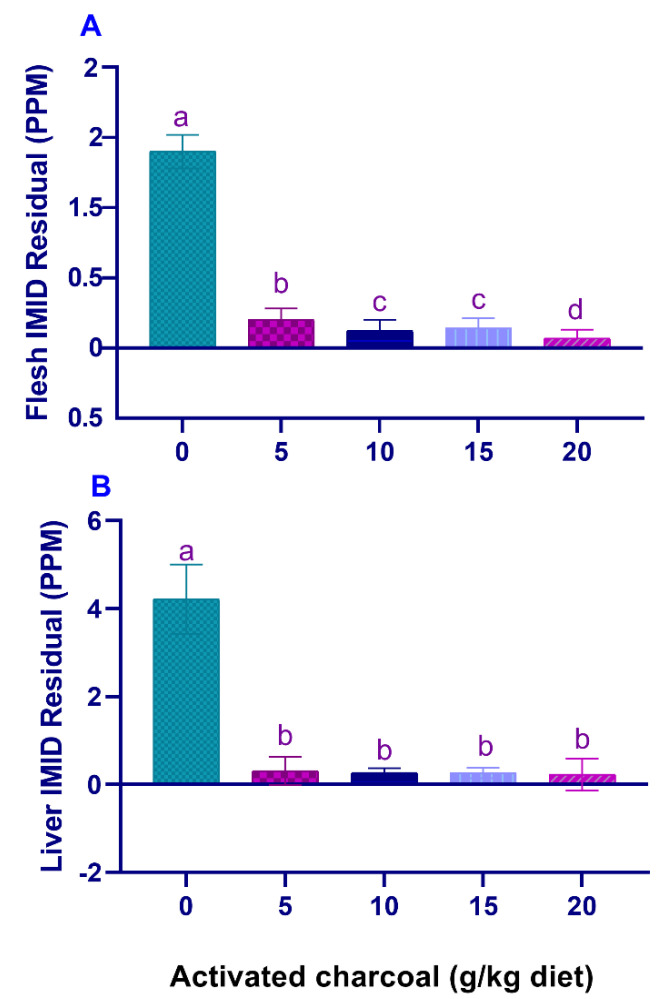
Residual imidacloprid (IMID) levels in the flesh (**A**) and liver (**B**) of Nile tilapia fed on diets supplemented with graded, activated charcoal (AC) levels for 2 months and then exposed to a sub-lethal IMID level for 2 weeks. (a, b, c, d) indicate significant differences between groups.

SR (%) and RPS (%) (Table 5) were significantly increased (*p* < 0.05), and IMID residual levels in the flesh (Figure 3A) and liver (Figure 3B) were significantly decreased (*p* < 0.05) in all AC groups compared with the AC 0 group after exposure to sub-lethal IMID toxicity for 2 weeks.

**Figure 4:**
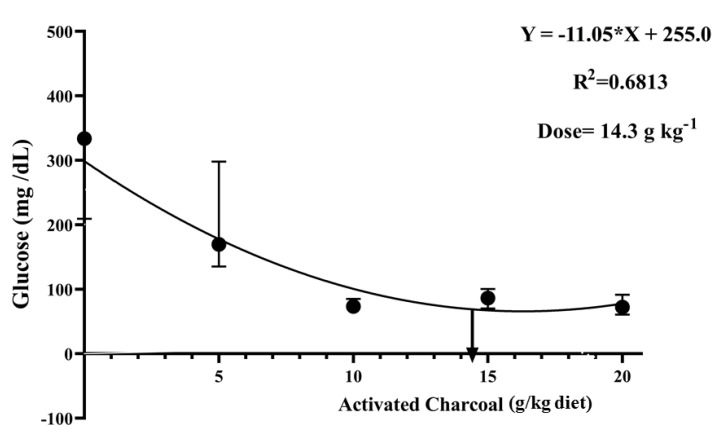
Second-order polynomial regression equation between blood glucose levels of Nile tilapia fed different dietary activated charcoal (AC) levels for 8 weeks and then exposed to a sub-lethal imidacloprid (IMID) level for 2 weeks. Values expressed as means ± S.E.M.

**Figure 5:**
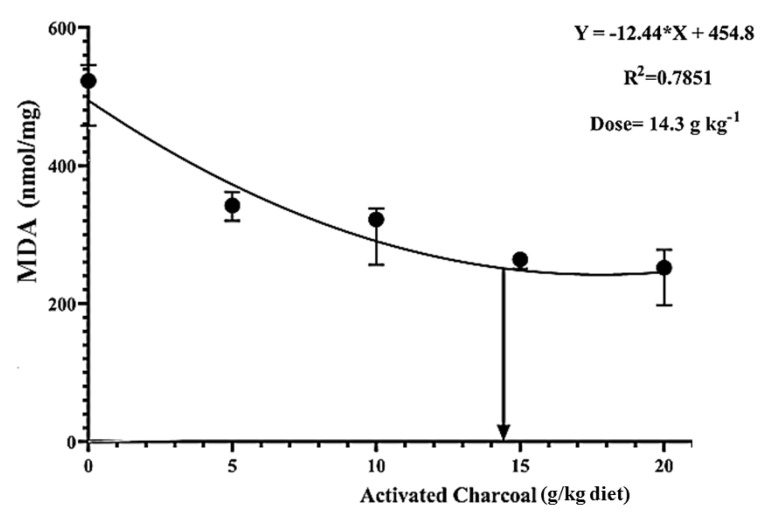
Second-order polynomial regression equation between malondialdehyde (MDA) levels of Nile tilapia fed different dietary activated charcoal (AC) levels for 8 weeks and then exposed to a sub-lethal imidacloprid (IMID) level for 2 weeks. Values expressed as means ± S.E.M.

There was a significant decrease (*p* < 0.05) in blood glucose (Figure 4) and serum MDA levels (Figure 5) in all AC groups compared to the AC 0 group after exposure to sub-lethal IMID toxicity. Second-order polynomial regression analysis showed that the lowest glucose and MDA levels (Figure 4 and Figure 5) were found at dietary supplementation with 14.30 g/kg diet after exposure to a sub-lethal IMID dose. This result suggests that dietary supplementation with 14.30 g/kg diet in the formulated feeds of Nile tilapia could mitigate the stressful effects of sub-lethal IMID toxicity.

## 4. Discussion

### 4.1. Experiment I-Feeding Trial

#### 4.1.1. Growth Performance

The present study reported significant enhancement of growth indices of Nile tilapia fed on diets supplemented with graded AC levels for 8 weeks. Pirarat et al. [9] illustrated that Nile tilapia fed on 2% AC-supplemented diet for 4 weeks showed noticeably improved growth performance and intestinal histomorphological criteria. Abdel-Tawwab et al. [10] illustrated that a 7.0 g/kg diet considerably enhanced the TFI and growth indices of Nile tilapia. Michael et al. [19] showed that 3% commercial wood charcoal improved the growth performance, nutrient utilization parameters, and proximate chemical composition of red tilapia juveniles. Dietary supplementation with a diet of 5 g of bamboo charcoal kg-1 noticeably enhanced the growth of flounder juveniles [16]. Moreover, dietary supplementation with charcoal and wood vinegar mixture at 5 and 10 g/kg diet for 8 weeks considerably increased the FER and WG of flounder [18]. However, Boonanuntanasarn et al. [2] reported no statistically significant differences in the growth of Nile tilapia fed diets supplemented with graded AC levels for 4 weeks. These inconsistencies may be associated with several factors, including different AC (source, composition, and supplementation levels), experiment (design, rearing, and period), and fish differences (species, IBW, and feeding habits).

The improvement of the growth performance of Nile tilapia in the current study may be explained by several factors such as (1) the adsorptive ability of AC, which helps in the elimination of the impurities and gases from the intestinal tract, which will, in turn, improve the digestion of diets [6,7], (2) dietary supplementation with AC, which could improve the absorptive functions of the intestinal villi, which will consequently increase the feed utilization [8], and (3) improving the intestinal histomorphometric parameters such as the intestinal villi height of Nile tilapia [2,9], and giant trevally [13,14]. The increase of the intestinal villus height will increase the surface area of absorption, contributing positively to the absorption of nutrients [53].

#### 4.1.2. Hemato-Biochemical Indices

Hematological profile could be considered important physiological bioindicators for evaluating the overall performances and health status of fish [54]. In the present study, there were no statistical differences in the RBC, PCV, Hb, and WBC count of Nile tilapia in all experimental groups. Our results were consistent with those reported by Boonanuntanasarn et al. [2], who reported no significant differences in RBCs’ count and Hb and hematocrit values of Nile tilapia fed AC-supplemented diets. Moreover, similar findings were reported in African catfish fed on AC-based diets [11]. In a similar sense, there were no differences in Hb and hematocrit values in Tiger puffer fish fed diets supplemented with bamboo charcoal [15]. These findings suggest that dietary AC levels did not negatively affect the health status of the treated fish.

On the other hand, a significant increase in TP, ALB, and GLO values was recorded in all AC-supplemented groups in the current study. Samadaii and Bahrekazemi [12] illustrated that a 15 g/kg diet significantly decreased the TP and ALB values of big sturgeon (*Huso huso*).

Transaminases are biomarkers of fish liver functions [36,55]. The increase of these enzymes in fish plasma is regarded as an indicator of liver damage after exposure to aquatic toxicants [56,57,58]. The results reported a significant decrease of ALP, AST, and ALT activities observed in all AC-supplemented groups, and these findings suggest a healthy status of the liver of the treated fish. Yoo et al. [18] found that dietary charcoal and vinegar mixture noticeably decreased ALT and AST activities in Olive flounder. A similar decrease in ALT and AST activities were also noticed in Nile tilapia fed AC-supplemented diets [10]. Moreover, dietary supplementation with 15 g/kg diet considerably decreases ALT and AST activities in big sturgeon [12]. Contrarily, there were no significant changes in ALT and AST activities in Nile tilapia fed AC-supplemented diets [2]. These differences may be attributed to different AC sources, supplementation levels, experimental design, rearing conditions, and study period.

#### 4.1.3. Immunity and Antioxidant Biomarkers

LYZ is an important enzyme in the non-specific immune responses of fish, required for breaking the cell walls of G+ve and G−ve bacteria [59]. Respiratory burst activity of fish phagocytes is associated with the attack of the challenged pathogens during the process of phagocytosis [60]. NO increases the ability of macrophages to engulf and destroy the challenged foreign pathogens [61]. In the current study, there were significant increments in serum LYZ, respiratory burst activities, and NO values in AC-supplemented groups, which indicate an enhancement of immune responses of the treated fish.

MDA levels are indicators of LPO, which occurs during the oxidative damage of host tissues due to the overproduction of reactive oxygen species [62,63]. A decrease in serum MDA levels in all AC groups suggests a decrease in LPO. Our results were inconsistent with those reported by Abdel-Tawwab et al. [10], who found no significant changes in the MDA levels of Nile tilapia fed AC-supplemented diets.

### 4.2. Experiment II-Responses to Sub-Lethal Imidacloprid (IMID) Toxicity

Reports showed the toxicological influences of IMID in different fish species [23,24,25,26,27,28]. Herein, it was found that after exposure to a sub-lethal IMID dose for 2 weeks, there were significantly increased SR% and decreased blood glucose, MDA levels, and IMID residual levels in the flesh and liver in all AC groups compared with the control group. These findings indicate that dietary AC can protect Nile tilapia against sub-lethal IMID toxicity and reduce its bioaccumulation in fish body. Moreover, dietary supplementation with 14.30 g AC kg^−1^ diet was the ideal dose to counteract the stress effects of IMID. In the same sense, Abdel-Tawwab et al. [10] found a significant decrease of MDA and glucose levels of Nile tilapia fed AC-supplemented diets after environmental heavy metals’ exposure. Moreover, a significant decrease in heavy metals’ bioaccumulation was also observed in tissues of big Sturgeon fed AC-supplemented diets compared to those fed the control diet [12]. Furthermore, Naiel et al. [28] demonstrated a significant decrease of IMID residual levels in flesh of Nile tilapia previously fed on diets supplemented with both ChNPs and vitamin C. Our recently published study showed that dietary supplementation with clinoptilolite and/or ChNPs significantly decreased the mortality rates and IMID residual levels in flesh of Nile tilapia [34].

These findings could be attributed to the characteristics of AC as a universal antidotal treatment of several toxicants and pollutants. The possible mechanisms of dietary AC could be related to several factors, including (1) its chelating properties, (2) increased elimination rate, and (3) increased adsorption to toxic elements, which will subsequently decrease the absorption of toxicants [2].

## 5. Conclusions

The findings of the present study indicated that dietary AC could enhance the growth performance and improve serum biochemical measurements, antioxidant capacity, and non-specific immunity of Nile tilapia juveniles. Moreover, a 14.30 g/kg diet could be recommended as an ideal dietary supplementation dose to mitigate the stressful effects of sub-lethal IMID toxicity in Nile tilapia.

## Figures and Tables

**Table 1:** Feed ingredients and proximate chemical composition of the commercially purchased basal diet (%) (on air-dry basis) (previously published in Abdelghany et al. [35]).

Feed Ingredients	(%)
Fish meal	15
Yellow corn	32
Soybean meal (44%)	20
Corn gluten meal (60%)	14
Wheat bran	13
Vegetable oil	4
Vitamin premix ^1^	1
Mineral premix ^2^	1
Total	100
**Proximate chemical composition (g/kg) as fed basis**	
Crude protein (CP) (*n* × 6.25)	311.5
Crude lipids (CL)	75.6
Ash	40.8
Crude fiber (CF)	53.7
Nitrogen free extract (NFE) ^3^	518.4
Gross energy (MJ per 100 g) ^4^	19.17

^1^ Composition (per kg): Manganese (53 g), Zinc (40 g), Iron (20 g), Copper (2.7 g), Iodine (0.34 g), Selenium (70 mg), Cobalt (70 mg), and Calcium carbonate (as carrier) up to 1 kg.^2^ Composition (per kg): Vitamin A (8,000,000 IU), Vitamin C (500 mg), Vitamin D3 (2,000,000 IU), Vitamin E (7000 mg), Vitamin K3 (1500 mg), Vitamin B1 (700 mg), Vitamin B2 (3500 mg), Vitamin B6 (1000 mg), Vitamin B12 (7 mg), Biotin (50 mg), folic acid (700 mg), Nicotinic acid (20,000 mg), and Pantothenic acid (7000 mg).^3^ NFE = 100 − (CP + CL+ Ash + CF).^4^ Calculated as 23.4 kJ g^−1^, 39.2 kJ g^−1^, and 17.2 kJ g^−1^ for protein, lipids, and carbohydrates, respectively.

**Table 2:** Growth parameters and survival rate (%) of Nile tilapia fed diets supplemented with graded, activated charcoal (AC) levels for 8 weeks.

Parameters	Experimental Groups					*p* Value
	AC 0	AC 5	AC 10	AC 15	AC 20	
IBW (g)	33.66 ± 0.05	33.22 ± 0.09	33.17 ± 0.06	33.14 ± 0.12	33.15 ± 0.32	0.356
FBW (g)	51.05 ± 0.08 c	55.35 ± 0.33 a	54.75 ± 0.25 a	54.44 ± 0.26 a	53.82 ± 0.61 ab	0.002
WG (g)	18.39 ± 0.98 b	22.13 ± 0.34 a	21.58 ± 0.24 ab	21.30 ± 0.76 ab	20.67 ± 0.63 ab	0.029
TFI (g)	30.33 ± 0.13 c	31.32 ± 0.92 b	33.19 ± 0.52 a	33.17 ± 0.02 a	31.29 ± 0.32 b	<0.001
FER (g/g)	0.55 ± 0.12 b	0.66 ± 0.02 ab	0.71 ± 0.02 a	0.68 ± 0.10 ab	0.66 ± 0.05 ab	0.003
FCR (g/g)	1.80 ± 0.25 a	1.50 ± 0.85 bc	1.41 ± 0.28 d	1.47 ± 0.37 c	1.51 ± 0.92 bc	0.011
SR (%)	100	100	100	100	100	0.476

IBW: initial body weight, FBW: final body weight, WG: weight gain, TFI: total feed intake, FER: feed efficiency ratio, FCR: feed conversion ratio, SR: survival rate. Values in the same row showing different letters are statistically significantly different (*p* < 0.05). Data are presented as the mean ± S.E.M.

**Table 3:** Hematological profile of Nile tilapia fed diets supplemented with graded, activated charcoal (AC) levels for 8 weeks.

Parameters	Experimental Groups					*p* Value
	AC 0	AC 5	AC 10	AC 15	AC 20	
**Erythrocyte constituents**						
RBCs (10^6^ × µL)	2.22 ± 0.19	2.53 ± 0.36	2.80 ± 0.44	2.61 ± 0.07	2.66 ± 0.39	0.593
Hb (g/dL)	8.80 ± 0.57	9.38 ± 0.79	10.20 ± 0.23	9.74 ± 0.95	9.67 ± 0.93	0.596
PCV (mg/L)	29.5 ± 1.44	32.1 ± 2.57	34.5 ± 1.44	32.6 ± 0.57	33.7 ± 3.33	0.568
MCV (fL)	134.6 ± 4.23	129.0 ± 2.33	123.4 ± 1.27	125.8 ± 1.75	128.7 ± 3.43	0.585
MCHC (g/dL)	29.75 ± 0.52	29.20 ± 0.21	29.63 ± 0.57	29.87 ± 0.41	28.72 ± 0.80	0.328
**Leucocyte constituents**						
WBCs (10^6^ × µL)	3.40 ± 0.17	3.68 ± 0.23	3.85 ± 0.87	3.64 ± 0.05	4.03 ± 0.03	0.068
Lymphocyte (10^6^ × µL)	1.34 ± 0.79 b	1.71 ± 0.72ab	1.83 ± 0.11ab	1.65 ± 0.38ab	2.01 ± 0.01a	0.002
Heterophils (10^6^ × µL)	1.15 ± 0.03	1.11 ± 0.31	1.20 ± 0.32	1.12 ± 0.10	1.22 ± 0.33	0.129
Eosinophils (%)	8.53 ± 0.09	7.07 ± 0.06	6.49 ± 0.29	7.69 ± 0.12	5.46 ± 0.23	0.199
Monocytes (%)	18.53 ± 0.02	16.30 ± 0.27	14.99 ± 0.17	16.48 ± 0.09	14.39 ± 0.43	0.588

RBCs: red blood cells, Hb: hemoglobin, PCV: packed cell volume, MCV: mean corpuscular volume, MCHC: mean corpuscular hemoglobin concentration, WBCs: white blood cells. Values in the same row showing different letters are statistically significantly different (*p* < 0.05). Data are presented as the mean ± S.E.M.

**Table 4:** Serum biochemical indices of Nile tilapia fed diets supplemented with graded, activated charcoal (AC) levels for 8 weeks.

Parameters	Experimental Groups					*p* Value
	AC 0	AC 5	AC 10	AC 15	AC 20	
**Blood protein profile**						
TP (g/dL)	3.03 ± 0.32 d	3.41 ± 0.26 c	5.01 ± 0.36 b	5.98 ± 0.19 a	5.74 ± 0.23 ab	<0.001
ALB (g/dL)	1.03 ± 0.05 d	1.77 ± 0.81 c	2.79 ± 0.28 b	3.93 ± 0.88 a	3.07 ± 0.66 ab	<0.001
GLO (g/dL)	1.34 ± 0.55 d	1.90 ±0.07c	2.23 ± 0.38 bc	2.06 ± 0.13 c	2.67 ± 0.29 a	<0.001
**Liver function enzymes**						
ALP (U/L)	13.82 ± 0.73 a	9.98 ± 1.05 b	7.11 ± 1.16 c	8.55 ± 0.49 b	7.28 ± 0.71 c	<0.001
AST (IU/L)	78.5 ± 1.75 a	36.5 ± 1.75 b	28.0 ± 1.69 b	31.0 ± 1.44 b	30.5 ± 1.02 b	<0.001
ALT (IU/L)	100.5 ± 1.56 a	90.6 ± 1.83 b	91.0 ± 1.97 b	66.5 ± 1.89 c	70.5 ± 1.88 bc	0.035

TP: total protein, ALB: albumin, GLO: globulin, ALP: alkaline phosphatase, AST: aspartate transaminase, ALT: alanine transaminase. Values in the same row showing different letters are statistically significantly different (*p* < 0.05). Data are presented as the mean ± S.E.M.

**Table 5:** Relative percentage survival and mortality rate (%) of Nile tilapia fed diets supplemented with graded, activated charcoal (AC) levels for 8 weeks and then exposed to a sub-lethal level of imidacloprid (IMID) for 2 weeks (Experiment II).

Table	Total No.	Dead Fish	SR (%)	MR (%)	RPS (%)
AC 0	32	6	81.25	18.75	-
AC 5	32	5	84.40	15.60	16.66
AC 10	32	2	93.75	6.25	66.66
AC 15	32	2	93.75	6.25	66.66
AC 20	32	2	93.75	6.25	66.66

SR: survival rate (%), MR: mortality rate, RPS: relative percentage survival.

## Data Availability

Available from the corresponding author on call.
